# The Global Health Watch

**DOI:** 10.1371/journal.pmed.0010003

**Published:** 2004-10-19

**Authors:** Mike Rowson, David McCoy, Amit Sen Gupta, Armando de Negri Filho

## Abstract

Why three non-governmental organizations are launching an alternative to the World Health Report

## Teaser

Mobilising civil society around an alternative World Health Report

At the World Health Assembly in May 2003, three civil society groups—the People's Health Movement, the Global Equity Gauge Alliance, and Medact—discussed the need for civil society to produce its own alternative to the World Health Organisation's World Health Report. We felt strongly that we needed to produce a global health report that had equity and the right to health at its heart. We also needed a way to monitor the performance of global health institutions themselves. The idea of an alternative to the World Health Report has developed into an initiative called the Global Health Watch, which we are launching next year.

## The Three Key Players

Medact (http://www.medact.org) is a United Kingdom–based global health charity, undertaking education, research, and advocacy on conflict, poverty, and the environment.

The Global Equity Gauge Alliance (http://www.gega.org.za) was created to participate in and support an active approach to monitoring health inequalities and promoting equity within and between societies. The Alliance currently includes 11 member-teams, called Equity Gauges, located in ten countries in the Americas, Africa, and Asia.

The People's Health Movement (http://www.phmovement.org) is a global network of activists, organisations, and social movements. Its goal is to re-establish health and equitable development as top priorities in local, national, and international policy-making, with comprehensive primary health care as the strategy to achieve these priorities.

## Why an Alternative Is Needed

Concerted action by civil society has had tremendous success in the field of international health—global grassroots campaigns on infant feeding, smoking, and drug prices have changed policies and people's lives.[Fig pmed-0010003-g001]

**Figure pmed-0010003-g001:**
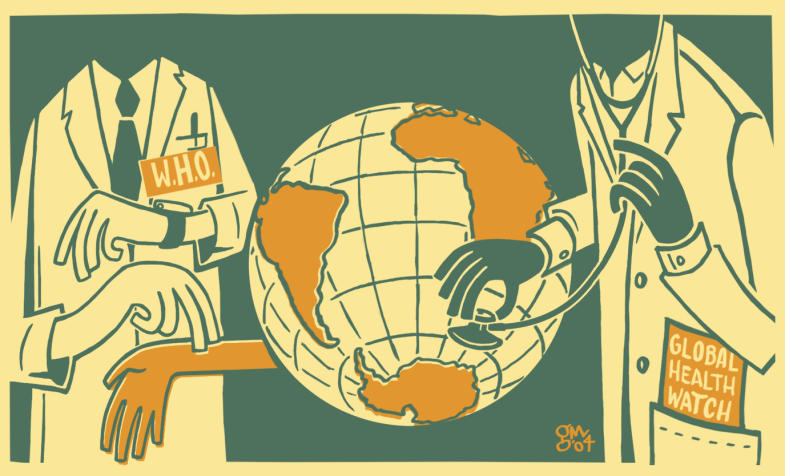
The Watch: Examining the world's health from an alternative perspective (Illustration: Giovanni Maki, Public Library of Science)

But over the last two decades—at the same time as these campaigns have scored victories—there has, in some parts of the world, been a stagnation and even reversal of the dramatic gains in life expectancy witnessed by many others for much of the 20th century. These reversals, unprecedented outside times of war and famine since the early 1800s and a scandal in a world of enormous wealth and technological prowess, have once more thrown the spotlight on how underlying social and economic problems affect health and health services.

The setbacks have also underlined appalling failures of health development policy. Ambitious targets to achieve ‘Health for All’ agreed to at the end of the 1970s by health ministers from around the world have failed miserably; a similar fate seems likely for the targets set out in the Millennium Development Goals for 2015. As a result, there are large question marks hanging over the effectiveness of international health policy.

These are the reasons why we have decided to produce Global Health Watch, which we hope will become a regular report on international health issues (Box 1). We believe that civil society campaigners need to look at the broader health agenda—beyond single-issue advocacy. Major concerns about health systems such as poor pay and working conditions for health professionals, creeping commercialisation, and plummeting public investment have not had the attention they deserve. Likewise, broader determinants of health—such as education, water, food, and the environment—are often insufficiently regarded when health policies are formulated. The Watch attempts to focus minds on the need for more integrated planning across sectors and on the creation of health systems that promote social justice rather undermine it.

**Box 1. Global Health Watch—2005 Report Contents**

**Section A: The Politics and Economics of Health in the 21st Century**

**Section B: The Health Care Sector**

- Health systems that promote social justice
- Responding to the commercialisation of health care
- The pharmaceutical industry, access to medicines, and intellectual property rights
- Human resources: the lifeblood of health systems
- Responding to HIV/AIDS
- Gene technology and the attainment of health for all
- Sexual and reproductive health

**Section C: Beyond Health Care**

- Environmental challenges
- Militarism and conflict
- Water
- The right to food: land, agriculture, and household food security
- Education

**Section D: Marginalised Groups**

- Indigenous peoples
- People with disabilities

**Section E: Monitoring of Institutions and Resource Flows**

- World Health Organisation
- World Bank
- World Trade Organisation and trade agreements
- Global Fund and Pepfar (United States President's Emergency Plan for AIDS Relief)
- Monitoring of international promises on aid and debt relief

**Section F: Summary and Strategies for Action**

## How Will the Watch Be Different?

This is how the Watch will be alternative: it will present options for health policy-makers that question the dominant reform agenda that emphasises market-driven and diseased-based approaches to health care. A policy bias against government action and a lack of creative thinking about how governments can shape health care markets to work in favour of equity and social inclusion are unfortunate features of global health debates. More recently, the emphasis has been placed once again on campaigns against specific diseases such as HIV/AIDS and tuberculosis, despite the universally acknowledged importance of building and maintaining health systems that can respond to the broader needs of patients.

We hope the Watch will present some alternative and imaginative thinking about how health services can respond creatively to the many challenges they face, with a strong focus on basic principles of equity and universality and avoiding top-down disease-focussed programmes that neglect the broader determinants of health. We have invited some of the most interesting and innovative thinkers in health policy—from both developing and developed countries and from academia and civil society—to help us achieve these objectives.

The Watch will also be ‘alternative’ in another sense—it will act as a regular monitor of the policies, governance, and funding of the institutions affecting global health, including the World Health Organisation and World Bank, something no other health report undertakes. We hope to offer proposals for reform, as well as to stimulate further action by civil society to make these institutions more accountable and responsive to the needs of the poor and vulnerable.

## Linking Civil Society Groups

It is important to say that the three networks and organisations that have convened the Watch are really just its initiators. In the end we hope the Watch will be backed by as many individuals, organisations, and social movements as possible, strengthening the links between civil society organisations across countries and across health-related sectors, and increasing the power and influence of the report itself. Already, many have expressed their interest in the project, and their willingness to contribute: through writing chapters, contributing case studies, and launching the Watch and promoting it in their country when it is finished. Groups from India and Brazil are planning parallel national Watches.

We plan to launch the Watch at the second People's Health Assembly, which will be held in Ecuador in July 2005. We don't want this report to be addressed just to health activists or health policy-makers or academics. If we are going to create change we need to capture the imagination of the broader health professional community and the public at large. That is why we encourage readers to get involved and tell others about the Watch and to use it to throw down a challenge to those who call the shots at national and international levels.

